# Joubert Syndrome Presenting With Oculomotor Apraxia and Motor Developmental Delay: A Case Report From a Neuro-Ophthalmology Clinic in Saudi Arabia

**DOI:** 10.7759/cureus.21638

**Published:** 2022-01-26

**Authors:** Rahaf A Mandura, Nawal A Arishi

**Affiliations:** 1 Ophthalmology, King Abdul-Aziz University, Jeddah, SAU; 2 Neuro-ophthalmology, Jeddah Eye Hospital, Jeddah, SAU

**Keywords:** prognosis, multidisciplinary approach, ocular abnormality, molar tooth sign, joubert syndrome

## Abstract

Joubert syndrome is an autosomal recessive genetic disorder that was first described in 1969. It can present with neonatal respiratory distress, ocular motility abnormalities, developmental delays, and other congenital cerebellar malformations. It is also connected to autism, hydrocephalus, and duodenal atresia. The incidence and severity of the disease are variable according to different presentations. We report a case of a female infant that was born to nonconsanguineous marriage and diagnosed at the age of four months with Joubert syndrome. The patient presented with global developmental delay and abnormal bilateral eye movements. Upon further investigation, brain magnetic resonance imaging showed a molar tooth sign, which is a characteristic finding and one of the diagnostic criteria of Joubert syndrome. A multidisciplinary team approach with ophthalmology, pediatrics, and physiotherapy departments was used, and the patient showed good progress in ocular, neurological and mental development. In conclusion, Joubert syndrome can be diagnosed early with the help of magnetic imaging and a multidisciplinary approach is necessary to provide good quality of life to these patients.

## Introduction

Joubert syndrome (JS) is a very rare autosomal recessive genetic heterogeneously inherited non-progressive disorder that was first described by Marie Joubert in 1969 [1]. It is characterized by neonatal hypotonia, congenital ataxia, global developmental delay, and at least one of the following features: neonatal respiratory distress and ocular motility abnormalities, including nystagmus and congenital oculomotor apraxia [2]. Other abnormalities that can be found with JS are facial dysmorphism, low-set ears, polydactyly, delayed speech, autism, meningoencephalocele, microcephaly, retinal dystrophies, kidney and liver diseases, soft tissue tumors of the tongue, and duodenal atresia [2]. It is accompanied by a congenital malformation of the brainstem and cerebellar vermis that composes the pathognomonic finding of a “molar tooth sign” that is evident on brain axial magnetic resonance imaging (MRI) [3,4].

Because of its rarity, there is little epidemiological data on JS but its prevalence has been estimated to be around 1:80,000 to 1:100,000 of live births [5]. The non-specific and variable clinical presentation of JS causes a delay in its diagnosis for several months after birth despite the presence of the clinical features in the neonatal period [6]. The average age for its diagnosis has been reported by Maria et al. as being 33 months [7]. The majority of children survive till adulthood and have an overall good prognosis [8]. In the Arab world, consanguineous marriages are common. However, JS incidence and mutation have been equally linked to consanguineous and nonconsanguineous marriages [9]. Early detection of this disease is vital to positively affect the outcome as appropriate intervention can be started earlier. Here, we present a case of JS in a nonconsanguineous family from Saudi Arabia.

## Case presentation

A three-year-old female child was born to nonconsanguineous marriage. The parents had been referred to genetic counseling; however, no genetic testing was conducted. The baby weighed 3.3 kg and was at full term in good and stable condition and an uneventful pregnancy course and delivery. The delivery was done by cesarean section due to previous multiple cesareans. She was the third child for a healthy 27-year-old mother and a healthy 35-year-old father. She has no history of neonatal respiratory distress, apnea, seizure or feeding difficulty, and no history of neonatal intensive care admission. She was discharged home with her mother in good general health. At the age of four months, the parents noticed that the child was unable to follow objects with abnormal movement of both eyes. Parents gave a history of developmental delay and the absence of neck holding or turning. There was no history of trauma and no family history of similar medical conditions.

On examination, there were no obvious dysmorphic features. However, the developmental delay was noted with no social smile in response to voices, she cannot support her head or grasp objects placed in her hands. Examination in our neuro-ophthalmology clinic revealed poor visual attention and inability to fixate or follow moving objects with both eyes tending to roll upward most of the time. Bilateral horizontal pendular nystagmus was quite obvious without extraocular muscle restriction or cranial nerve involvement. Reactive pupils without relative afferent pupillary defects were noted. Anterior segment examination was normal. Fundus examination revealed clear media, normal optic disc, macula, and retinal vessels.

**Figure 1:**
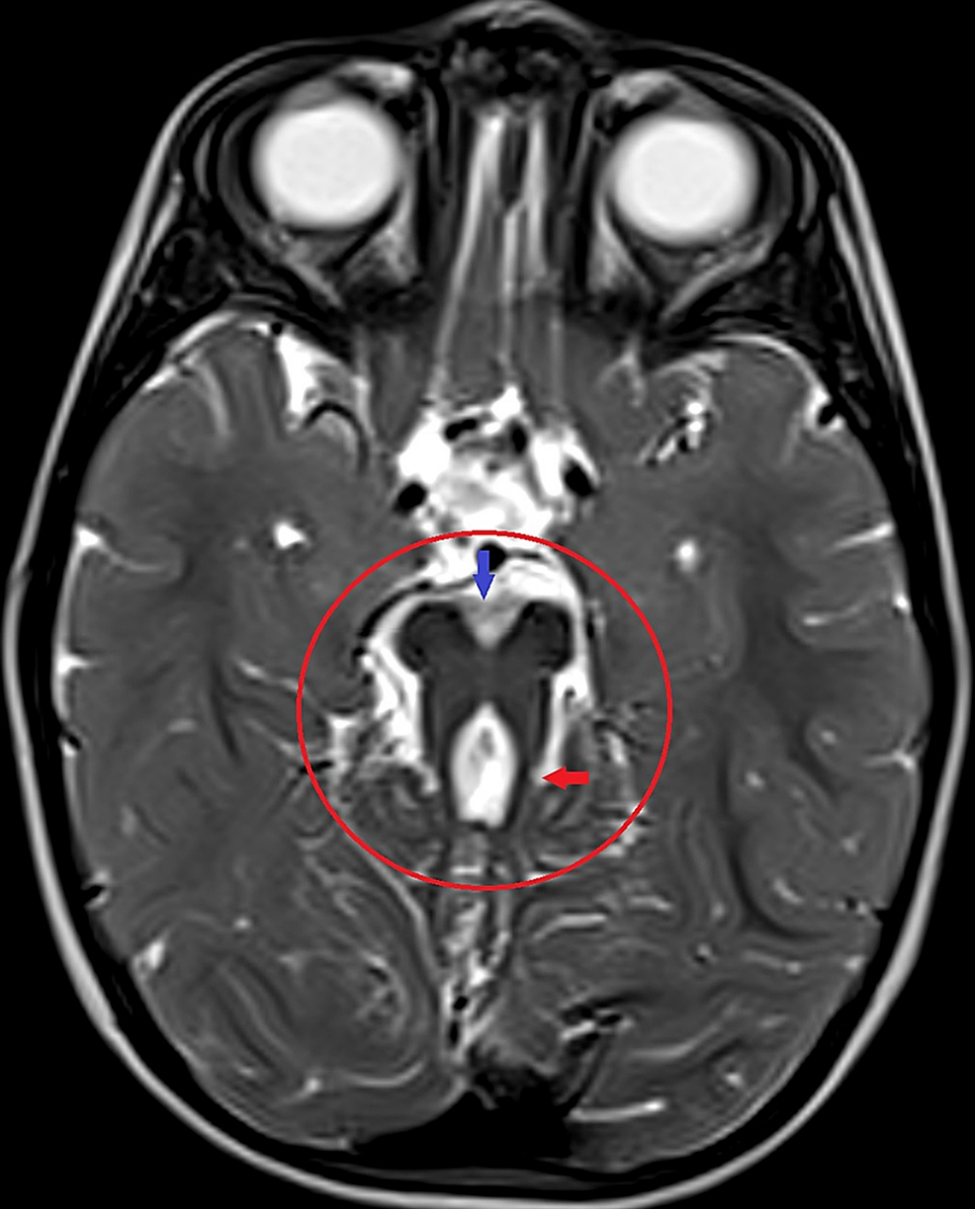
Axial view of brain magnetic imaging resonance showing molar tooth sign (red circle), deep interpeduncular fossa (blue arrow) with thick and elongated superior cerebellar peduncles (red arrow).

**Figure 2:**
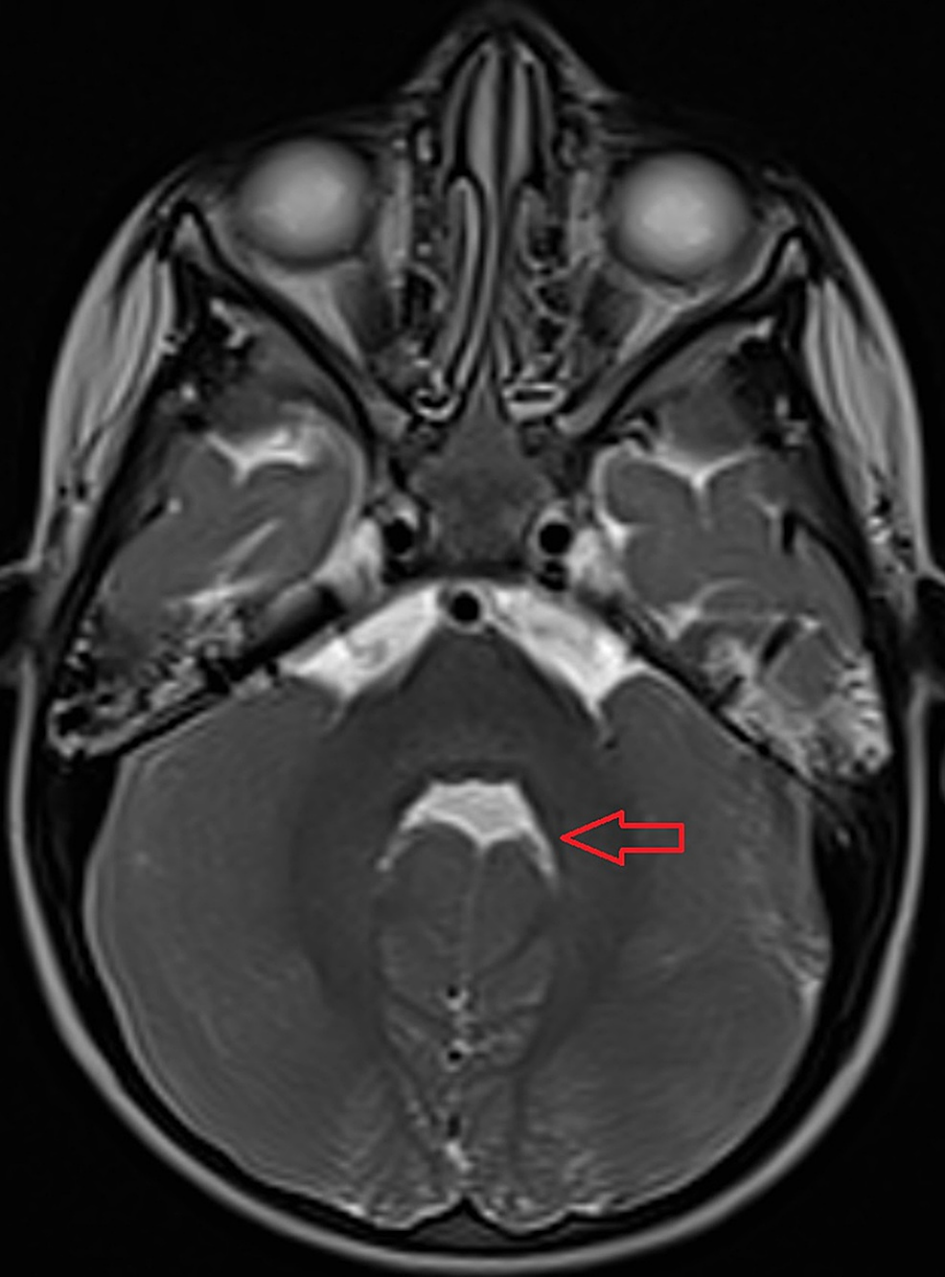
Axial view of brain magnetic resonance image showing absence of the cerebellar vermis results in a bat-wing shaped fourth ventricle (red arrow).

As a result, MRI brain was arranged and revealed prominent, thickened, and elongated superior cerebellar peduncles with deep interpeduncular fossa showing molar tooth sign (Figure 1) and hypoplastic cerebellar vermis (Figure 2), which is a classical pathognomonic finding of JS. Therefore, the child was diagnosed with JS based on clinical and MRI findings and was referred to the pediatrics department to rule out systemic associations of JS. Afterward, the patient showed normal cardiovascular, pulmonary and abdominal examination. Moreover, chest x-ray, echocardiography, and abdominal ultrasound were all normal and the patient was cleared from any systemic associations. However, neurological examination revealed hypotonia with normal tendon reflexes.

Extensive counseling for the parents was done and the patient was followed with a multidisciplinary approach with the ophthalmology, pediatric neurology, behavior and development, and physiotherapy department. Following the subsequent years, she showed improvement of visual attention with the ability to fixate and follow objects with the disappearance of the nystagmus. However, she was still showing evidence of motor and speech developmental delay that is lagging behind the appropriate milestones for a normal child of her age. On examination at the age of three years, she was looking well and alert with no dysmorphic features but showed a tendency to keep her mouth open with tongue protrusion. She can stand on her own and walk for a short distance with cerebellar ataxia and unsteady gait and can say some separated words. On ocular examination, visual acuity showed good fixation and the following ability without nystagmus. She demonstrated oculomotor apraxia with difficulty in changing fixation. The change in fixation was accomplished by a head thrust that overshot the target and was followed by a rotation of the head backward in the opposite direction once the fixation is established. Furthermore, pupils’ examinations were normal in both eyes. Orthoptic examination showed normal eye alignment without strabismus. Extraocular muscle examination showed a full range of movement in both eyes. Cycloplegic refraction was +2.75 -0.50 x10 in the right eye, and +2.00 -1.00 x180 in the left eye. Fundus examination showed normal optic disc, macula, and retinal vessels without pigmentary changes in both eyes. No glasses prescription was needed, and the parents were advised to attend the regular follow-up appointments and to continue the multidisciplinary approach.

## Discussion

JS belongs to an extensive group of inherited disorders with an autosomal recessive pattern of inheritance; however, a few studies have also reported an X-linked recessive pattern of inheritance in some families [10]. JS is caused by mutations of the cilia which are subcellular organelles that have an essential rule in signal transduction during embryonic development which is referred to as ciliopathies [11]. Other diseases in this group include cystic kidneys, retinal degeneration, intellectual disability, infertility and skeletal alterations [11]. Mutations in genes associated with JS can be found in up to 90% of patients with this condition. The various clinical presentations are accounted to the multiple genetic mutations [12]. Mutations in nine genes encoding cilia have been identified, which attribute to 50% of mutations in JS-related disorders [13]. INPP5E (JBTS1), AHI1(JBTS3), NPHP1(JBTS4), CEP290 (JBTS5), TMEM67/MKS3 (JBTS6), RPGRIP1L (JBTS7), ARL13B (JBTS7), and CC2D2A (JBTS9). Mutations in the AHI1 (JBTS3) gene and CEP290 (JBTS5) account for 7%-10% of JS, whereas mutations in the NPHP1 (JBTS4) gene cause approximately 1%-2% of Joubert syndrome. Impaired vision due to retinal dystrophy occurs in both AHI1 (JBTS3) gene and CEP290 (JBTS5); however, CEP290 (JBTS5) and NPHP1 (JBTS4) with this genetic mutation develop a progressive kidney disease called nephronophthisis and renal cortical cysts [12,13]. The incidence of JS is estimated to be 1:80,000 and 1;100,000 [14].

The diagnostic criteria of JS include the molar tooth sign, which can be seen on axial views from cranial MRI studies. It is comprised of three findings including hypoplasia/aplasia of the cerebellar vermis, deepening of interpeduncular fossa, and the presence of thick elongated superior cerebellar peduncles. Other diagnostic criteria also include intellectual impairment or developmental delay and hypotonia in infancy. Moreover, neonatal respiratory distress and ocular motility abnormalities are findings that can support the diagnosis if one or both of them was found clinically. Motility abnormalities are comprised of nystagmus and/or congenital oculomotor apraxia related to saccadic dysfunction [13].

Saccadic function, typically with head thrusts or turns, primary position nystagmus, which are usually see-saw, pursuit abnormality, and retinal findings are suggestive of dystrophy. They are the most common ophthalmic phenotypic features of JS found according to Khan et al. in a cohort study conducted on eight patients in Saudi Arabia [15]. Similarly, Maria et al. described eye findings in 13 patients with JS and reported that the most consistent findings were poor vestibule-ocular reflex cancellation, defective saccades, and impaired smooth pursuit [16]. Other findings included strabismus, retinal dystrophy, nystagmus (gaze-holding and pendular), optic nerve dysplasia, periodic alternating gaze deviation, and chorioretinal coloboma. In our patient, the most characteristic ophthalmic findings were oculomotor apraxia due to saccadic dysfunction and primary position pendular nystagmus.

The pathogenesis of saccadic and pursuit dysfunction which is the most distinctive feature in JS is attributed to cerebellar atrophy; however, these abnormalities cannot be localized because there are many other regions of the brain that regulate saccades and thus potentially may be involved, for example, the frontal eye fields, parietal lobe, basal ganglia, and brainstem [17]. The retina is one of the organs most frequently involved in JS, in the form of retinal dystrophy, due to progressive degeneration of photoreceptor cells [16]. Severity ranges from congenital retinal blindness to progressive dystrophy with variable vision [16]. Furthermore, coloboma involving the retinal pigment epithelium, neurosensory retina, and choroid are known associations of this syndrome as well [18]. However, in our patient, there were no retinal, choroidal or optic nerve abnormalities.

The presence of molar tooth signs is considered variable and can dramatically affect the prognosis of patients with JS [19]. According to Brancati et al., the presence of molar tooth signs can be accompanied by other central nervous system problems such as hydrocephalus, hypothalamic hamartoma, and the absence of the pituitary gland. Patients reported with such malformations have higher chances of developing epilepsy and occipital (meningo) encephalocele [19]. Furthermore, the prognosis of JS is also dependent on the severity of the respiratory distress and breathing dysregulations found soon after birth. Comparably, our patient did not have any sort of respiratory distress and was not admitted to the hospital after birth as the first presentation occurred after the fourth month. In such cases, a multidisciplinary approach is very beneficial in alleviating the quality of life of those patients and making their developmental progress up to their age.

## Conclusions

JS is a rare autosomal recessive ciliopathy which is a complex multi-organ group of diseases. It is characterized by hypoplasia of cerebellar vermis and elongation of cerebellar peduncles causing the pathognomonic imaging finding of molar tooth signs. The variable disease presentation can range from a mild abnormality in eye movement that improves with age to severe retinal degeneration that causes blindness at birth. Delayed diagnosis is usually attributed to its non-specific presentation. Therefore, physicians should be aware of the peculiar clinical and radiological findings of JS that help in early diagnosis with a multidisciplinary team approach and management to enhance the outcomes.
